# Eagle eyed or bird brained?

**DOI:** 10.1038/s41433-023-02568-y

**Published:** 2023-06-23

**Authors:** David Williams

**Affiliations:** St John’s College Cambridge, Cambridge, CB2 1TP UK

**Keywords:** Neuroscience, Scientific community, Medical research

## Abstract

The importance of the visual system to birds for behaviours from feeding, mate choice, flying, navigation and determination of seasons, together with the presence of photoreceptors in the retina, the pineal and the brain, render the avian visual system a particularly fruitful model for understanding of eye-brain interactions. In this review we will particularly focus on the pigeon, since here we have a brain stereotactically mapped and a genome fully sequenced, together with a particular bird, the homing pigeon, with remarkable ability to navigate over hundreds of miles and return to exactly the same roosting site with exceptional precision. We might denigrate the avian species by the term bird brained, but here are animals with phenomenal abilities to use their exceptional vision, their eagle eyedness, to best advantage.

## Abstract (zh)

鸟类的视觉系统在进食、择偶、飞行、导航和季节判定等行为方面至关重要, 加上视网膜、松果体和大脑中存在光感受器, 使鸟类的视觉系统成为理解眼-脑交互作用的一个富有成效的模型。在这篇综述中, 我们聚焦于信鸽这种特殊的鸟类, 因为我们有其大脑立体定位图和完整的基因组测序, 并且信鸽具有能够导航数百英里, 并精确地返回原栖息地的能力。我们使用“鸟脑”这个词可能会低估鸟类, 但这些动物有着鹰一般的眼睛, 和能充分利用它们独特的视觉系统的非凡能力。

## Introduction

**Fig. 1:**
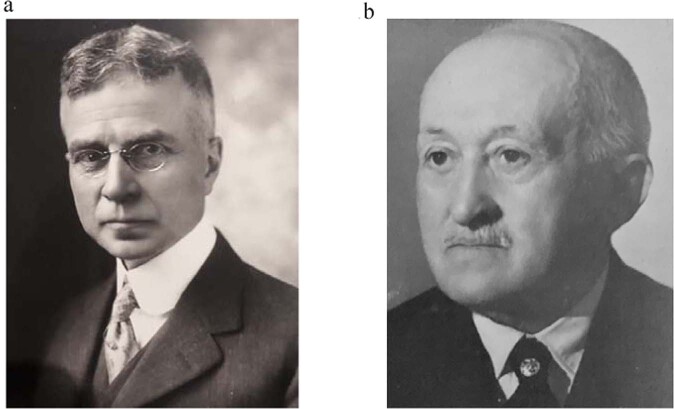
Two near contemporaneous ophthalmologists. **a** Casey Albert Wood. **b** André Rochon-Duvigneaud.

May I start by introducing you to two near contemporaneous ophthalmologists from some time ago – Casey Albert Wood (1856–1942) (Fig. 1a) and André Rochon-Duvigneaud (1863–1952) (Fig. 1b) both of whom devoted their lives to the study of comparative ophthalmology, producing major works on avian ophthalmology, respectively *The Fundus Oculi of Birds* [1] and *Les Yeux et le Vision des Vertébrés* [2]. For both, study of the remarkable vision of birds held promise of improving human vision. Consider a bird of prey hovering high over a motorway verge, able to see a rodent on the ground far below with far better visual acuity than ours [3] up to ten times the acuity relative to body size [4]. Or a passerine flying through a heavily wooded area without ever impacting the smallest of branches. What a flicker fusion frequency (FFF) its retina must be able to achieve! The answer is 145 Hz for collared flycatchers, three times our FFF [5]. Birds see not only without three cone photoreceptors but in ultraviolet also and can navigate on migratory routes by detecting polarised light from the sky above and magnetic fields from the earth below far beyond our detection ability in the electromagnetic spectrum. They determine seasonal timings using extra-retinal photoreceptors allowing them to breed at just the right time to optimise feeding opportunities for their young after hatching. In all these functions the link between eye and brain is critical. In the context of the theme of this symposium, *The Eye and the Brain*, what can avian vision tell us about the interactions between the organ detecting and that interpreting what is seen?

Consider this diagram of the avian head, from Wood’s magnum opus (Wood 1917, Fig. 2). The volume of the eyes is considerably greater than the volume of the brain. How different from that of the human head where the eyes are tiny compared to the cerebrum.

**Fig. 2:**
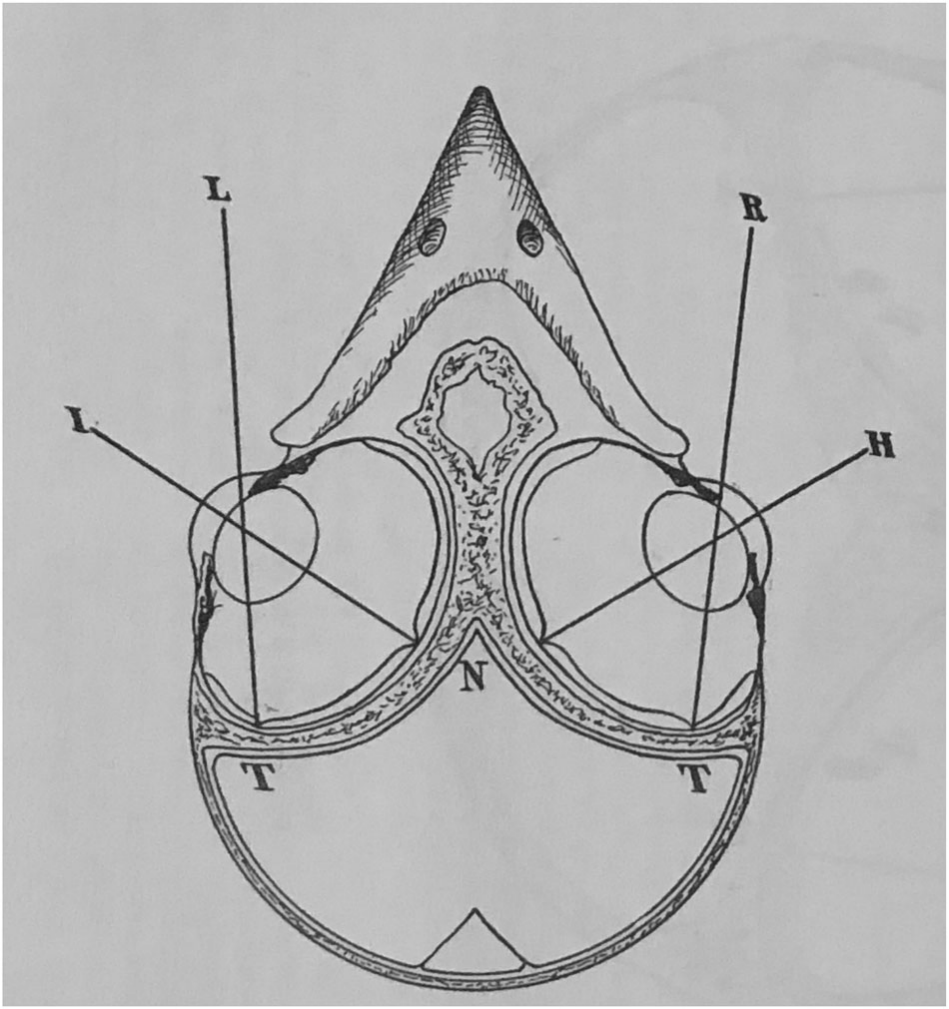
Diagram of the avian eyes and skull showing the large size of the globes compared with brain. From Wood *The Fundus Oculi of Birds* 1917.

As Rochon-Duvigneaud would have put it, the bird is ‘a wing guided by an eye’ [2]. But if that is the case where does the brain fit in? We say that someone with limited intelligence is bird-brained but for the size of their brains, birds, and especially corvids, are exceptionally able to perform executive functions to the extent that Clayton and her colleagues have termed crows and jays ‘flying apes’ [6]. Is this because the avian brain is a model in miniature of the human brain or do birds employ a different set of processes in vision-related decision-making?

A significant problem with using the avian visual system as a model for the mammalian one is that functionally similar neuronal circuits have very different anatomies. Another remarkable comparative ophthalmologist was the American physiologist Gordon Lynn Walls with his remarkable *The Vertebrate Eye and Its Adaptive Radiation* [7]. He suggested that mammals had undergone a ‘nocturnal bottleneck’ adapting to a predominantly nocturnal lifestyle in competition with diurnal pterosaurs and dinosaurs during the Mezozoic era. Mammals lost cone photoreceptor opsins and non-visual photoreceptor opsins together with developing anatomical adaptations promoting on the one hand sensitivity to low luminance environments and on the other binocular stereopsis. Birds however retained exceptional diurnal vision.

Other major drivers of avian vision exist in addition to guiding flight. Determining the position of the beak and control of its movement are key in feeding as, in some birds, is position of the feet for prey capture. Detection of a potential predator is also a more or less constant activity for most birds. Reproduction is clearly a central factor in survival of the species and visual tasks in mate choice are crucial. Here the avian eye as significantly superior to the human – much mate choice relates to visual signals in the ultraviolet, a range of the visual spectrum to which our eyes have no access [8]. Determination of seasonality is key in defining when to mate, ensuring that chicks hatch when food is plentiful. The onset of mating is generally determined by day length in most bird species. John Hunter collected and dissected sparrows throughout the year and showed a variation in testicular size [9]. Appreciation of light intensity must be required to determine daylength. As long ago as 1935, however, Benoit showed that blind ducks still showed a seasonal change in gonad size [10] and that light shone directly onto the brain via thin glass rods could provoke reproductive behaviour in these birds even after enucleation [11]. Here was early evidence of extraocular photoreception, although work on minnows detecting pigmentation in conspecifics without the need for eyes was performed more than twenty years earlier [12].

## Ocular anatomy

**Fig. 3:**
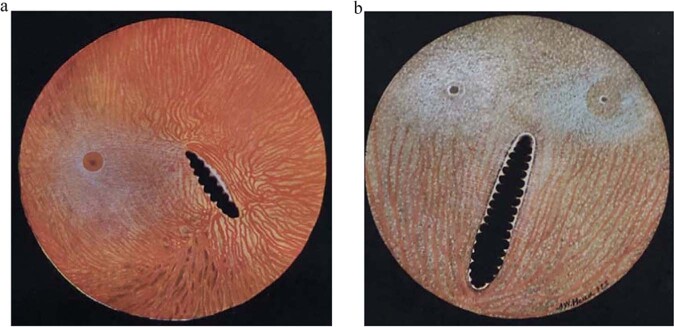
The fundus of the Tawny owl and the Short-tailed hawk. The black pleated pecten in both with one fovea in the Tawny owl (**a**) and two in the short-tailed hawk (**b**), both pictures From Wood *The Fundus Oculi of Birds* 1917.

The avian globe is large in relation to the rest of the skull and in species with tubular eyes the projected image is correspondingly larger than for an oblate globe. The size of the cornea is large allowing for optimal light passage into the eye also occasioned by the large lens with its wingwulst allowing attachment of the muscles of accommodation. The retina is avascular with nutrients and oxygen provided by the pecten, a pleated eminence of the choroid lying in the posterior vitreous and beating from side to side as the eye moves allowing oxygen and nutrients to reach the entirety of the posterior segment (Fig. 3) [13]. The fovea, so crucial in obtaining the highest possible acuity, is deep, indeed convexiclivate in several raptorial species [14], and many have two foveola, though quite how the brain interprets information from both is somewhat difficult to investigate, with two modes of vision, monocular and binocular [15]. A key worker seeking to join evaluation of neural networks and complex avian behaviour was Professor Barrie Frost who showed with single cell microelectrode recordings on the tectal surface of the bifoveolate kestrel, that the central monocular fovea, with emmetropic or hypermetropic vision, views more distant objects while the temporal binocular fovea with a myopic focus just below the beak views closeup objects during interaction with prey items [16]. Having said that other workers have suggested that the foveola are used to guide raptors in the logarithmic spiral path they take to apprehend a prey item [17]. Evaluation of avian neurobiology requires collaboration between neuroanatomists and behaviourists.

## Visual pathways

There are two prime ascending visual pathways from retina to brain in the bird, the thalamofugal and tectofugal, corresponding to the geniculo-striate and the colliculo-pulvinar-cortical pathways in mammals. The tectofugal pathways plays the greatest part in avian high level vision while it is the geniculo-striate in mammals. The cellular organisation of neural circuitry is substantially different as well, for while in mammals the circuits are arranged in layers in the neocortex [18], the avian nuclear pallium is composed of nuclear clusters [19].

The thalamofugal visual pathway includes a retinal projection to the geniculatis pars dorsalis of the thalamus in which a full 10% of optic nerve axons are involved in calculating distance to collision with an oncoming object. The avian telencephalon is divided into two regions first the wulst and secondly the dorsal ventricular ridge (DVR). The wulst is characterised by a laminated structure unique to birds within which the dendrites of neighbouring neurons stay to the specific layer rather than traversing between different layers as they do in the mammalian visual cortex. The specific functions of each cluster are strongly connected anatomically but spread throughout the pallium. Although this seems very different from the mammalian cortex, the entopallium, the visual area of the nidopallium, exhibits neural markers similar to the mammalian neocortical layer 4 while the sensory processing area of the DVR is characterised by neurons similar to those found in the mammalian deep neocortex. The nidopallium is the focus for further high-level sensory tasks was part of a circuit with the basal ganglia.

The tectofugal visual pathway comprises 90% of the optic nerve axons projecting to the optic tectum, a large structure when compared to bird’s reptilian relatives with 15 different layers involved in orientation and spatial awareness much like the mammalian equivalent the superior colliculus.

## Visual tasks

Birds are capable of significant cognitive tasks even with their so much smaller cranial capacity compared to primates and cetaceans. We have so many different avian species, from passerines like sparrows to raptors like eagles, that reviewing their visual neurobiology would take a whole book such as the excellent volume edited by Zeigler and Bischof, though the field has progressed considerably since its publication in 1993 [20]. Here we will concentrate on the pigeon with both a stereotactic atlas of the brain on the one hand [21] and full genome sequencing on the other [22].

Pigeons have been reported to perform visual tasks as diverse as on the one hand distinguishing between paintings by Monet and Picasso [23] and on the other evaluating pathological and radiological images of breast cancer [24]. While such visual tasks are remarkable, determining how the eye and brain work to achieve such visual feats is taxing. Single neuron recordings have shown the areas involved in motion detection in the thalamofugal system as noted above, and particularly time to collision calculation, vital in a flying bird. Understanding form perception is considerably more taxing than documenting motion detection but as social behaviour in many birds such as pigeons is so important determining what visual cues are key in conspecific interactions and how they are recognised is particularly valuable in understanding eye-brain coordination. Using video screens with computer generated stimuli to detect how pigeons interacted virtual birds with enlarged, reduced or obliterated facial features showed that preference was exhibited to birds with enlarged beaks [25]. Facial recognition does occur in birds [26] but studies seeking to identify cells or area—patches—of neurons in the avian brain specifically recognising avian faces have, to date not been successful [27].

More relevant to the bird is homing for which the pigeon is renowned, and phenomenal feats of migration in other avian species. The Eastern lark sparrow which makes flights of thousands of miles to return to the same field year after year is just one example [28]. Such complex tasks as image recognition or migration cannot easily be explained by a simple evaluation of nerve circuits.

Using video screens with computer generated stimuli to detect how pigeons interacted virtual birds with enlarged, reduced or obliterated facial features showed that preference was exhibited to birds with enlarged beaks. Facial recognition does occur in birds and imaging in crows with functional magnetic resonance imaging (MRI) has demonstrated brain areas active in recognising human faces, and differentiating between aggressive and caring expressions [15]. Studies seeking to identify cells or areas—patches as we would call them in primates—of neurons in the avian brain specifically recognising avian faces have, to date not been successful [27]. Perhaps we are looking in the wrong areas, at the wrong species or indeed for cells reacting to the wrong signals?

## A genomic perspective

Here the homing pigeon shows its value. Darwin realised that the breeding of pigeons was an ideal example of evolution [29], part of ‘an experiment on a gigantic scale’ [30]. The homing pigeons has been bred over centuries from the rock dove to develop it’s homing instinct. What can comparison between the genomes of the homing pigeon and other closely related but non-homing con-specifics tell us? Population genomic analyses comparing homing pigeons with feral rock pigeon showed 163 positively selected genes. These could of course be genes encoding excellent flying ability but three genes in particular were noteworthy. Lipoprotein related protein 8 (LRP8 otherwise known as ApoER2), Arginine vasopressin receptor 1 A (AVPR1A) and Single-stranded DNA-binding protein 3 (SSBP3). The first two are specifically important in learning and memory, but may not tell us anything about the involvement of visual function in homing. LRP8 encodes a protein essential in longterm potentiation, central in learning and memory [31, 32]. In mice lower LRP8 expression is associated with defective spatial memory [33]. A point mutation in the gene for LRP8 in homing pigeons changes lipid affinity, altering hippocampal pathways and enhancing memory [34]. Given our interest in the link between brain and eye, maybe this gene is rather more brain than eye. The same may be the case with AVPR1A, a gene linked in rodents with social recognition where olfaction is key [35] and in humans with musical memory when hearing predominates [36]. Indeed it seems that non-visual clues may be more important that ophthalmic ones in long-distance homing, although pigeons rendered anosmic certainly recall visual landmarks in homing [37].

## Polarised light and magnetic fields

It may be that polarised light plays an important part in avian navigation through the sun compass as may the celestial compass [38] or sensing of the earth’s magnetic field in night-migrating birds [39]. Indeed one may calibrate the other [40]. The mechanism by which photoreceptors, and particularly the unusual double cones of the avian retina, sense the earth’s magnetic field would appear to be by radical pair magnetoreception. Transitions from singlet to triplet states of radicals induced by photo absorption by cryptochromes, a type of flavoprotein, are induced and, once formed, are influenced by magnetic fields [41, 42]. The recent finding of cryptochrome gene expression in the retinas of homing pigeons [43] and gene polymorphisms associated with homing performance [44], together with structural analysis showing how magnetic fields influence photochemical reactions [45] validates research fifty years ago showing that magnets interfered with pigeon homing [46].

Quite how the changes resulting from differential gene expression in the brain and the retina of these homing pigeons interact is unclear. The hippocampal formation is substantially larger in homing pigeons compared with that of non-homing breeds but one has to ask whether this is a function of genetics or behaviour. London taxi drivers have larger hippocampi than bus drivers but this relates to their driving experience rather than being an innate genetic predisposition for taxi-driving!

We have known for some time that homing pigeons use magnetoreception as part of their homing navigation [47] with iron-binding proteins and magnetite playing important roles in electron signal generation in sensing geomagnetic fields. Cryptochromes generate radical pairs which yield photo-induced electron transfer reactions, these occurring in orbit, nasal cavity and upper beak and wattle, these known to be larger in homing pigeons that other breeds from the time of Darwin and before [48]. The flavoprotein glutathione disulphide reductase exists in high levels in the double cones of the homing pigeon retina [25], showing that the retina does much more than merely detect photons!

## Light detection by the brain

Having said that the converse can be true. There are other areas that can detect photons and respond to them apart from the retina. In the same way as we know light sensitive ganglion cells occur in the mammalian retina, photoreceptors are to be found in the hypothalamus and pineal gland of birds [49, 50], these particularly related to detection of changes in daylength, vital in determining both commencement of mating behaviour and of migration [51]. Birds rendered blind as a result of a mutation in the retinal guanylate cyclase-1 gene and a head mask to prevent cranial extraocular light detection synchronised their feeding behaviour to a light dark cycle of >12 h [52] while in enucleated birds brain illumination through the skull synchronised the birds’ behaviour, though pinealectomised birds only synchronised at high light intensities [53]. Clearly, in the chicken at least, a multiple photoreceptor system involving retinal, pineal and other deep brain photoreceptors is important in defining and controlling many behaviours key to the birds’ life histories.

## Conclusion

This close association of photoreceptors in the brain as well as in the eye, together with the importance of the visual system in the bird in feeding, mate choice, flying, navigation and determination of seasonality, makes the bird an ideal choice for a model of eye-brain integration. It is hoped that this brief review will stimulate both biologists and ophthalmologists to further collaborations defining further the interactions between eye and brain in these remarkable species.
